# The Evaluation of the Efficacy of a Novel Subunit Vaccine in the Prevention of *Cryptosporidium parvum*-Caused Diarrhoea in Neonatal Calves

**DOI:** 10.3390/ani15020132

**Published:** 2025-01-08

**Authors:** Martine Reijnders, Mark van Roosmalen, Hans Holtslag, Susan Arts, Suzanne Pel, Divine Dufé, Marianne Kaashoek, Geert Vertenten, Tine van Werven, Steven Sietsma, Christophe Roy, Nicolas Herman

**Affiliations:** 1 MSD Animal Health, 5831 AN Boxmeer, The Netherlands; mark.roosmalen@merck.com (M.v.R.); hans.holtslag@merck.com (H.H.); susan.arts@merck.com (S.A.); suzanne.pel@merck.com (S.P.); divine.dufe@merck.com (D.D.); marianne@kaashoek.com (M.K.); 2 Universitaire Landbouwhuisdieren Praktijk (ULP), Faculty of Veterinary Science, University of Utrecht, 3481 LZ Harmelen, The Netherlands; t.vanwerven@uu.nl (T.v.W.); ssietsma@ulp.nu (S.S.); 3 Clinique Vétérinaire des Mazets, 15400 Riom es Montagnes, France; roille@orange.fr (C.R.); nicolas.herman@gmx.fr (N.H.)

**Keywords:** *Cryptosporidium parvum*, cryptosporidiosis, vaccine, neonatal calf scours, clinical study, passive immunisation, colostrum, serum

## Abstract

*Cryptosporidium parvum* (*C. parvum*)-caused calf diarrhoea (scours) is widespread and leads to calf mortality in cattle industries worldwide. A recent discovery of a glycopeptide epitope on the *Cryptosporidium* parasite recognized by a monoclonal antibody has led to the development of a new vaccine. It was designed for cows to pass on passive immunity to their neonatal calves. This vaccine has been demonstrated to be safe and efficacious in pre-clinical studies. In the process of marketing registration, two clinical studies were conducted with this vaccine on 16 commercial dairy and beef farms collecting data, assessed by the European Medicine Agency. In these studies, its effectiveness in the field was evaluated. The natural challenge and clinical disease caused by *Cryptosporidium parvum* in the calves in these studies was, however, not high enough to show a significant reduction of most of the clinical signs, although all trends were in favour of the vaccination groups. Nonetheless, the duration of the diarrhoea episodes of the dairy calves was significantly shorter in the vaccination group. Additionally, the levels of Gp40 antibodies in the blood of the calves was notably higher in the vaccination groups, demonstrating the principle of passive immunization. Overall, this vaccine, which can be used concurrently, non-mixed with Bovilis^®^ Rotavec^®^ Corona, introduces an additional approach to mitigate the impact of *C. parvum* infections in neonatal calves.

## Simple Summary

*Cryptosporidium parvum* is a parasite that causes severe diarrhoea in newborn calves, often leading to death, which significantly impacts cattle industries globally. To address this problem, a new vaccine has been developed to help protect calves by vaccinating cows, making them generate antibodies that are passed to their calves through colostrum. These antibodies help reduce the severity and duration of diarrhoea in the calves. Studies conducted on commercial dairy farms in the Netherlands and beef farms in France showed that calves from vaccinated mothers showed trends of fewer, less severe and significantly shorter episodes of diarrhoea. The vaccine also markedly increased the levels of specific protective antibodies in both the mothers’ colostrum and the calves’ blood. This new vaccine, which can be used alongside another common cattle vaccine, provides a new immunological addition to the set of tools used for reducing the burden of calf scours. It brings a new means of improving the health of newborn calves, benefiting the cattle industry and ensuring better livestock welfare.

## 1. Introduction

Dairy and beef producing farms worldwide face serious problems managing calf scours (diarrhoea). It is one of the most common illnesses, an important reason for treatments and a main cause for mortality in pre-weaned calves. Among several organisms causing neonatal calf scours, the four more frequently identified pathogens are *Cryptosporidium parvum* (*C. parvum*), rotavirus, coronavirus and *Escherichia coli* [1]. Bovine cryptosporidiosis is widespread [2,3,4] and causes calf mortality in many countries throughout the world [5,6,7]. Besides affecting animals, *Cryptosporidium* poses a public health threat through transmission between livestock and humans [8,9].

As *Cryptosporidium* infections manifest in young calves, with clinical signs appearing in the second week of life when infections result in enteritis, scours and the shedding of large numbers of oocysts [10,11,12], preventive measures need to reach efficacy as soon as possible after birth. The prevention of rotavirus-, coronavirus- and *E. coli*-caused diarrhoea in calves is possible through vaccination via the passive immunization route, but for the reduction and control of cryptosporidiosis, farmers rely on meticulous young stock management with firstly the aim of optimising calf health and increasing resilience and secondly taking biosecurity measures [3]. Maintaining these highly hygienic measures on the farm is extremely difficult, as infected calves shed *Cryptosporidium* oocysts abundantly and these oocysts are insensitive to numerous disinfectants [3]. The only therapeutics available for *Cryptosporidium*-diseased calves are halofuginone lactate and paromomycin, with variable results [13]. No prophylactic measure like vaccination against *Cryptosporidiae* was available of yet [14]. In this report we present the results of the pivotal clinical studies conducted to obtain EMA (European Medicine Agency) marketing authorization for this newly developed vaccine targeting *Cryptosporidium parvum*.

Recently, a glycopeptide epitope recognized by a protective *Cryptosporidium* monoclonal antibody was found [15]. A vaccine containing this antigen was developed for the passive immunisation of newborn calves. This subunit vaccine is designed to generate high levels of specific antibodies in the colostrum of cows and heifers through vaccination in the last 2 months of pregnancy. When this colostrum is ingested by their newborn calves, it increases their specific immunity and reduces disease (i.e., diarrhoea and subsequent dehydration and depression) caused by *C. parvum* infection [16]. The vaccine has shown its safety and efficacy profile under controlled (laboratory) conditions [16] in the MSD AH Boxmeer R&D department. Subsequently, to collect field data for the marketing authorization of this vaccine, two clinical studies conforming to GCP (Good Clinical Practice) were conducted to be assessed by EMA. These studies are presented in this paper.

The European authorities state that when satisfactory efficacy has been documented in the context of pre-clinical studies, evidence of the effectiveness of particular administration methods under conditions of field use may also be acquired by showing that animals vaccinated under field conditions develop an appropriate immune response to the vaccination [17]. The objective of these field studies was to confirm the appropriate immune response, i.e., vaccine take, demonstrated via Gp40 antibodies in the colostrum and blood of dams and calves, respectively. Additionally, as supplementary data, an effort was made to validate the findings from the pre-clinical calf challenge studies with clinical findings in the field. Besides these efficacy parameters, the overall safety of the vaccine under typical usage conditions in the dams was evaluated. These studies were conducted on European commercial farms with confirmed *C. parvum* infection pressure and a documented history of clinical signs caused by cryptosporidiosis in neonatal calves. Any safety issues in the dams were reported to evaluate the safety of the vaccine and relevant clinical data were collected to assess if in these studies, clinical efficacy in the field could be demonstrated.

The target animals for this vaccine are dairy as well as beef cattle. As colostrum management is different in both farming systems, these studies were conducted in commercial dairy farms in the Netherlands as well as beef/suckling farms in France to confirm the effectiveness of the respective processes of passive immunization in the field.

## 2. Materials and Methods

All data were collected in compliance with the principles of the International Cooperation on Harmonisation for Veterinary Medicines (VICH) Good Clinical Practice (GCP). Study authorisation was obtained in the countries where the studies were conducted (the Medicines Evaluation Board, Amsterdam, The Netherlands and ANSES, Fougères, France). The owners gave written informed consent for their animals to be included in the study. The Investigators conducting these studies were veterinarians experienced in clinical studies and farm tending veterinarians who were trained for the purpose.

### 2.1. Vaccine

The vaccine investigated was not registered for marketing purposes at the time of these studies, but at present is known as Bovilis Cryptium^®^ (MSD Animal Health, Boxmeer, The Netherlands), a subunit vaccine designed to generate high levels of antibodies against *C. parvum* Gp40 antigen in cows and heifers to provide early passive immunization in calves when these ingest this antibody-rich colostrum short after birth.

On the enrolment day (study day 0 between September 2020 and January 2021), all eligible animals present on the farms were clinically examined and, if healthy and between 10–6 weeks before calving, included in the study. The study group (test or control 1:1) was allocated sequentially according to a n-Query 7.0 software-generated randomisation list for two treatment groups, stratified by site. Bovilis Cryptium^®^ (test group) or 0.9% saline solution (control group) was applied subcutaneously (SC) in the left side of the neck at 2 mL per dose. Bovilis^®^ Rotavec^®^ Corona was injected intramuscularly (IM) in the right side of the neck. Both vaccines were used according to their (proposed) SPCs (Summary of Product Characteristics). The animals were revaccinated with Bovilis Cryptium^®^ or re-injected with a saline solution 4 weeks later (see Table 1). A single-use syringe and a hypodermic needle were used. The animals were observed for immediate reactions to the vaccination. The administrator and assessor of the animals were different persons, to maintain blinding.

### 2.2. Selection of Farms

The studies were conducted on representative European farms with confirmed *C. parvum* infection pressure and a documented history of clinical signs caused by *C. parvum* infections, as confirmed by PCR tests in neonatal calves. The recent cases were tested by Rainbow test (Bio-X, Rochefort, Belgium [18]). The herds on these farms had a confirmed vaccination record to reduce the prevalence of scours caused by rota-, coronavirus or *Escherichia. coli*. Following this screening, eight dairy farms in the Netherlands and eight beef farms in France were selected.

The dairy farms in these studies were typical Dutch dairy farms which housed on average 160 dairy cows (Holstein Friesian breed) with an average production of 9200 kg milk/305 days. The typical period between calving on these farms was 400 days and the age at first calving was 24 months. Cows were all housed in a free-stall stable with cubicles and matrasses. Calves were separated from their dams shortly after birth and were fed colostrum and transition milk from their own dam only in their individual cubicles or hutches. Dairy cows were fed grass, maize silage, concentrate and additives. All herds were at least routinely vaccinated with Bovilis Rotavec Corona and some herds were also vaccinated against IBR and BVD.

The beef or dual-purpose herds selected for this study were in the west and massif central parts of France. The breeding cows included in the study were of the Charolais, Rouge Des Prés and Salers breeds. The farms had on average 92 dams in their herd, with a range from 33 to 235. The Charolais and Rouge Des Prés herds were kept in free stalls in winter and were turned outside in summer. The Salers were kept in tie stalls in the winter season and were turned outside in the mountains in summer. Both systems had seasonal calving. In all systems, the cows calved in separate calving boxes and were confined with their newborn calf for at least the first 8 days, with the calves all suckling their own dam, or the calves were housed individually and fed via assisted feeding from their own dam.

### 2.3. Inclusion Criteria Study Animals

The animals enrolled in these studies were healthy cows and heifers between 10 and 6 weeks before the expected date of calving. The exclusion criteria were any appearance of disease or unknown status of pregnancy. Calves born from study dams were automatically included in the study, but were excluded in cases where they received colostrum from any dam other than their own.

In the Netherlands, 8 dairy farms were selected for study activities. After the selection of the eligible cows and heifers, 295 animals were included between the 23 September 2020 and the 20 January 2021. The number of animals included per farm is presented in Table 2 below. These cows or heifers were between 1.7 and 12.1 years of age at vaccination. A total of 147 cows were allocated to the control group and 148 to the test group. The cows were vaccinated at the time they were between 10 and 6 weeks before the expected date of calving and ultimately received their first vaccination between 13.3 and 4.9 weeks before the actual date of calving in the control group and between 11.4 and 4.3 weeks in the test group.

In France, a total of 304 pregnant beef or dual-purpose cows and heifers between 1.8 and 15.9 years of age (78 Charolais, 21 Rouge Des Prés and 205 Salers) on 8 farms were included in the study between the 16th of October and the 14th of December 2020. The number of animals included per farm is presented in Table 3 below. Following the randomisation, 152 study animals were allocated to the control group and 148 to the test group. The animals were vaccinated for the first time between 13.0 and 4.3 weeks before the actual date of calving in the test group and between 21.6 and 4.6 weeks in the control group.

In the Netherlands, one cow from the test group died before calving and in France four animals were not vaccinated for the second time as the calves were born earlier than expected. These calves were withdrawn from the study. Additionally, four cows did not attribute to the efficacy data as one was sold (test group), one appeared not pregnant (control group) and two (control group) gave birth to a dead calf.

### 2.4. Medications and Therapies

The study treatment consisted of Bovilis Cryptium^®^ (MSD Animal Health) in the test group and saline solution in the control group. Both study groups were vaccinated with Bovilis^®^ Rotavec^®^ Corona (MSD Animal Health), aiming to eliminate the clinical effects of other diarrhoea causes as much as possible. Cows and heifers did not receive any other veterinary products at the same time as the study treatments and their calves were not treated with products active against *Cryptosporidium* (e.g., halofuginone lactate or paromomycin) during the first 21 days of life except in cases of serious veterinary need. In these cases, treatment took place after obtaining a faecal sample to test for scours causes with the Rainbow scours test [18]. All treatments applied in the study were recorded per individual.

### 2.5. Variables and Clinical Observations

The purpose of these studies was to compare the antibody levels in calf serum and detect any reduction of the incidence, severity and/or duration of diarrhoea the first 21 days after birth in calves born from and having ingested colostrum from vaccinated dams compared to calves from unvaccinated dams. The differences between the control and test group were measured by scoring their general health, faecal consistency and feed intake. The faecal consistency score (FCS) was described as 0–3, ranging from 0 to 3 according to the Madison–Wisconsin calf health score depicted in Table 4 [19].

General health was assessed using a score range from 0 (healthy and attentive) to 3 (signs of severe disease). Feed intake was scored 0 (readily drinking) to 2 (does not drink).

The primary clinical parameter was the incidence of calves with a faecal consistency score ≥ 2 during the observation period (21 days). The secondary parameters were the severity of diarrhoea based on the level of the scores and the duration of diarrhoea episodes, defined by the number of days a calf scored at least 2 for faecal consistency, and the overall clinical signs, summarized in the Total Clinical Score (TCS), which was calculated as the sum of the daily faecal consistency scores and the general health and feed intake scores. Additionally, all morbidities and any medications used were recorded. From these medications, the number of scours treatments was compared between both study groups. In case of mortality, a necropsy was performed to determine the cause of death.

To confirm the antibody response to vaccination, colostrum samples of all dams taken within 24 h postpartum were tested for antibodies against the Gp40 protein of *C. parvum*. The passive transfer of antibodies was tested by analysing the calves’ serum sample. Cows were clinically examined before and after vaccination. Notable local reactions were reported. The outcome of calving was registered.

### 2.6. Colostrum Management

The colostrum intake of the calves was monitored and recorded. In the dairy herds, shortly after calving (day 0), the colostrum of the cow was collected and at least 3 litres were fed to the new-born calf as soon as possible, optimally within 4 h after birth. On day 1, at least 1 litre of colostrum was fed and on day 2, 3 and 4, at least 0.5 litres were fed. Farmers were free to feed larger volumes if desired, but not less than the volumes described. When applicable, the colostrum and transition milk of the 2nd and later milkings was stored and fed in order of collection on the subsequent days to reach the minimal volumes.

In the beef herds, for the first four to eight days after birth the calf stayed either confined with its own mother or was housed with other calves and was assisted in drinking from its own dam 2–3 times a day. The first time the calf was seen drinking the estimated colostrum intake (categorised: enough, insufficient or none) was registered.

All calves in the studies were allowed to receive colostrum exclusively from their own dam.

### 2.7. Collection Colostrum and Serum

A soon as possible after giving birth and at least within 24 h, a colostrum sample from the study dams was collected. From the calves, the Investigator obtained one blood sample when the animals were between 3–17 days of age. This sample was of approximately 10 mL in volume, taken in a plain serum tube from the jugular vein.

### 2.8. Laboratory Analyses

The serum and colostrum were tested in the Intervet International BV R&D Boxmeer Laboratory. A specifically developed ELISA test was used to detect the presence of antibodies against the Gp40 protein. The antibody titre (in log2) was calculated with CBA (Calculation of Biological Assay) according to the Abend Vertical (Inside Interpolation) method. Samples were diluted using standard dilutions in the lab with cut-off values of <8.8 log2 and >19.9 log2. For calculation purposes these values were changed into, respectively, 8.7 log2 and 20.0 log2 in the final database.

Faecal samples of calves with Fc score ≥ 2 were tested on the same day of sampling with the Rainbow scours test BIO K 306 (Bio-X Diagnostics, Rochefort, Belgium).

### 2.9. Statistical Analysis of the Dataset

Sample size calculations were performed based on results from pre-clinical studies.

There was no need to use inferential statistics to analyse the serological data of the colostrum and serum. Descriptive statistics were used to present the data.

The faecal consistency score as well as the episodes of diarrhoea (duration of FCS ≥ 2) for each animal were compared between the test and the control group using generalised estimating equations (GEEs). The overall health status of the calves compared between study groups was analysed by comparing the Total Clinical Score using a non-parametric method: the Wilcoxon rank-sum test. The total number of days with FS > 2 was compared between groups using a mixed ANOVA model with farm as random effect to compare the duration of diarrhea episodes between the Test and Control groups.

The statistical analysis was performed at the 5% level of significance using SAS 9.4 (SAS Institute Inc., Cary, NC, USA).

## 3. Results

### 3.1. Demographic Data Calves

In the Netherlands, a total of 305 dairy calves were born from the study animals. Some calves died prematurely or were excluded from the analysis (due to post-partum mastitis and a lack of colostrum of their dams or because of being born before the second study vaccination could take place), leaving 283 calves, 143 calves in the control group and 140 calves in the test group, for clinical efficacy data analysis.

In the suckling calves from the French beef herds, an additional 305 calves were born. Due to early deaths or protocol violations, like when they were adopted by another dam or their dam was not fully vaccinated, 22 calves were subsequently excluded from the dataset. This left 283 suckling calves eligible for the analysis database with 144 in the control group and 139 in the test group. There were thirty-five dairy calves and five suckling calves which did receive colostrum from their own mother, but reportedly not enough. Despite this deviation from the protocol, the data from these calves were still used in the analysis as this is considered worst-case data, as in the given cases, a low colostrum intake is the expected course of events in the field.

### 3.2. Clinical Scores

#### 3.2.1. Incidence of Diarrhoea

The incidence of diarrhoea, specified as occurrence of faecal consistency scores ≥ 2, was low in the dairy calves and even lower in the suckling calves. Therefore, statistically significant differences were not seen between the study groups. In Holstein Friesian (HF) dairy calves, the daily percentage of calves with diarrhoea was the highest between 7–12 days of age, at up to 33.8% in the control group and 28.7% in the test group on day 10. In the control group, the odds ratio of having diarrhoea was 25% higher compared to the test group (odds ratio 1.25 [95% C.I.: 0.98, 1.61], *p*-value = 0.0770) (non-significant *p*-value). Percentages were summarized in Table 5.

In suckling calves, the overall incidence of calves with diarrhoea scores ≥ 2 was very low in both study groups, with 7.7% and 6.5% of calves having diarrhoea in the control and test group, respectively. The occurrence of diarrhoea in the control group was therefore not statistically significantly decreased, but the odds ratios show that the occurrence of diarrhoea was 12% more likely in the control group compared to the test group (odds ratio 1.12 [95% C.I.: 0.71, 1.77], *p*-value = 0.6147). Percentages are summarized in Table 6.

#### 3.2.2. Duration of Diarrhoea

In the dairy calves, the duration of the diarrhoea period was significantly shorter in the test group compared to that of calves in the control group (*p*-value = 0.0300). The calves in the control group showed diarrhoea for 2.2 days, while in the test group the diarrhoea episodes lasted for 1.8 days on average, as presented in Table 7.

In suckling calves, the average duration of diarrhoea periods was too short to show any difference; the average diarrhoea episode in both study groups was 0.6 days, as presented in Table 8.

#### 3.2.3. Severity of Diarrhoea

For the severity of diarrhoea, the level of the diarrhoea scores was assessed. In the control groups from both the dairy and suckling herds, there were more animals with a maximum FC score 3 (severe diarrhoea). In the dairy calves, 30.1% of the calves in the control group had a faecal consistency score of maximum 3 at least once, versus 22.1% in the test group. It was calculated that the chance of observing severe diarrhoea was 8% higher in the control group than in the test group (odds ratio 1.08 [95% C.I.: 0.86, 1.35], *p*-value = 0.5074). In the suckling calves, the chance of observing a high (2 or 3) faecal consistency score was 10% more likely in the control group (odds ratio 1.1 [95% C.I. 0.76–1.60], *p*-value = 0.5971). Both differences were not statistically significant. The distribution of the calves and their maximum scores is presented in Table 9.

#### 3.2.4. *Cryptosporidium* Infections

From calves with FCS ≥ 2, i.e., calves with diarrhoea, a faeces sample was obtained. In both studies, *C. parvum* was the pathogen most frequently found in these samples. In the calves from vaccinated dams, the percentage of *C. parvum*-positive samples was lower than in the control group. In the dairy study, the percentage of calves having at least one positive test result was 52.4% (75 calves) in the control group and 40.7% (57 calves) in the test group. In the control group of the suckling calves, 19 calves tested positive, which is 13.2% of the total number of calves in the control group. In the test group, only 10.7% (15 calves) was *C. parvum*-positive. The distribution of scours test results for *C. parvum* is presented in Table 10. In several samples, the Rainbow test was positive for multiple pathogens. Some calves were tested more than once. The most prevalent combined infection was *C. parvum* combined with *Clostridium perfringens*.

#### 3.2.5. Total Clinical Score

In both the dairy as well as suckling calves, the percentage of calves with a TCS > 0 was lower in the test group compared to the control group, although not statistically significantly. The percentage of dairy calves with an abnormal TCS (>0) was 88.8% (*n* = 127) in the control group compared to 85.7% (*n* = 120) in the test group (Wilcoxon rank-sum test *p*-value = 0.1778). In suckling calves, the percentage of calves with an abnormal TCS (>0) was 54.9% (*n* = 79) in the control group and 49.6% (*n* = 69) in the test group (Wilcoxon rank-sum test *p*-value = 0.4474).

### 3.3. Morbidity and Treatments

A total of 89 (61.4%) of the dairy calves in the control group and 77 (52%) in the test group were treated against clinical illnesses. All treatments and indications were listed for completeness. A variety of indications was listed, but most of the administered treatments were against diarrhoea, of which the percentage of treated calves was also higher in the control group (58.6%) than in the test group (49.3%).

The overall numbers of treated calves were much lower in the French breeding herds, but also in these herds, the percentage of calves treated against diarrhoea was higher in the control group (18.1%) than in the test group (15.1%). A total of 39 (27.1%) calves in the control group and 38 (27.3%) from the test group received medication. Most of the administered treatments were related to diarrhoea, followed by the number of treatments for omphalitis. The numbers of treated calves with indications are shown in Table 11. More than one indication for treatment was possible.

### 3.4. Mortality

In the dairy study, six calves died: three calves from the test group and three from the control group. These calves died of causes not related to the vaccinations of their dams like dystocia, congenital defects, and pneumonia and one calf from the control as well as one calf from the test group died with *Cryptosporidium*-positive diarrhoea.

A limited number of suckling calves died during the study: one calf from the control group died at an age of 8 days of diarrhoea caused by *Cryptosporidium*. One calf from the test group died on study day 21 from the clinical complications of omphalitis.

### 3.5. Antibody Titres in Colostrum and Calf Serum

The antibody levels against Gp40 were clearly higher in the vaccinated dams and their offspring compared to the controls. In dairy cows, the average log2 antibody titre measured in the control group was 14.9 ± 1.5 and in the test group 19.6 ± 0.8. In beef herds, the average log2 antibody titre measured in the control group was 15.7 ± 1.8 and in the test group 19.5 ± 1.2.

In the serum of the calves, the average log2 antibody titre measured in the control group was 11.3 ± 1.6 in dairy calves and 12.1 ± 2.0 in the suckling calves. In the test group this was 18.1 ± 1.7 in the dairy calves and 17.2 ± 2.4 in the suckling calves. These results include all calves in the study, among which were the 40 animals that were reported to have taken in less than the amount of colostrum aimed for by the farmers. Table 12 below shows the average colostrum and serum Gp40 titres.

### 3.6. Safety and Postvaccination Adverse Reactions

The vaccinated cows and heifers were reported to be healthy after vaccination and gave birth to calves as normal on the farms. The number and aspect of the local reactions after vaccinations were comparable between the Bovilis Rotavec Corona and Bovilis Cryptium. The number of treatments in the (vaccinated) adult animals was not elevated and indications for treatment were in line with treatments normally given in the transition period, i.e., mostly related to dry-off (all dairy cows), mastitis or retained placenta. One animal in the control group aborted. In total, seven control and thirteen test cows gave birth to a stillborn calf (defined as a calf born dead or dying within 24 h after birth). The numbers of stillborn calves were not higher than expected and the Investigators attributed these deaths in most cases to asphyxia after dystocia. Post-mortem examination of the other stillborn calves rendered causes like congenital malformations in the heart, atresia ani, aspiration pneumonia or enteritis. None of these causes can be attributed to the vaccination of the dams.

## 4. Discussion

These studies were conducted to obtain marketing registration of a newly developed vaccine. The confirmation of the everyday use of the vaccine and the practical execution of the passive immunization route under normal field circumstances was the first aim of these studies. Secondly, supportive clinical data were collected. The effectiveness of the passive immunization route was confirmed. From the clinical observations, the duration of diarrhoea episodes in the calves from vaccinated dairy dams was significantly reduced. Other clinical parameters, like the incidence of diarrhoea and severity of the diarrhoea scores, all showed positive trends but were not statistically significant. To demonstrate a reduction of clinical signs, animals require adequate levels of infection rates and seriousness of clinical signs. In the field studies described here, the clinical signs were not serious enough to show significant reductions in the test group.

It is noted that our findings emphasize the intricate nature of identifying significant differences between groups of calves affected by a multifactorial disease like calf scours. The interplay of genetic, environmental and management factors, coupled with the heterogeneity of animal populations and the temporal dynamics of the disease, all contribute to the complexity. Although these studies in the suckling calves started in winter, when animals are kept indoors and infection rates are normally at the highest, the clinical signs of scours in these studies were less severe than seen in previous seasons. The conduct of these studies themselves might well have added to these low manifestations of scours problems as a large part (est. 50%) of the herd was vaccinated, possibly reducing *C. parvum*-caused clinical signs overall.

When assessing the results of the serological tests in these studies, the dams as well as the calves in the test group showed markedly higher antibody levels against Gp40 in their colostrum or serum, respectively, demonstrating that vaccination increases the antibodies in the colostrum of vaccinated dams in the field and that, if this colostrum is taken in well by their calves, this results in increased antibody levels in their serum. These results corroborate previously reported experimental findings in which the newly developed vaccine was found efficacious after challenging neonatal calves with *C. parvum* in our pre-clinical studies [16].

When we focus on the treatments available for cryptosporidiosis, the results of our studies also align with many studies that examined the effectiveness of halofuginone lactate [20,21,22,23]. These studies found varying outcomes, likely also due to the impact of the multifactorial features of cryptosporidiosis. In these studies, the improvement in diarrhoea scores was not consistently observed in the treated groups and although the faecal oocyst counts were frequently reduced, complete elimination was not achieved.

Following these studies, it would be interesting to explore if the effect of vaccination could be assessed in settings with higher infection pressure. It would also be helpful to take new study parameters into account like cost effectiveness in relation to animal performance [24].

Finally, it has been shown that it is safe to administer Bovilis Cryptium^®^ concurrently (non-mixed) with Bovilis^®^ Rotavec^®^ Corona. This makes it possible to combine several preventive measures in the face of treating and preventing calf scours and to alleviate the burden calf health management puts on farmers. In addition to this, given the fact that the use of paromomycin is widespread as a treatment for cryptosporidiosis in calves, vaccination and improving specific immunity can reduce the need for antibiotic interventions, promoting more sustainable and responsible farm management practices.

## 5. Conclusions

The vaccine assessed in these studies caused no safety issues and specific antibody levels were markedly higher in the calves from vaccinated dams. These antibodies have proven to be effective in pre-clinical studies. Furthermore, in our field studies it was demonstrated that vaccination caused a significant reduction in the duration of diarrhoea episodes in dairy calves. Due to the unexpectedly low infection pressure of *C. parvum* on many of the study farms however, other clinical parameters only showed positive trends.

These results indicate that, by providing a means to supply young calves with specific protective antibodies, Bovilis Cryptium^®^ introduces a ground-breaking new approach and an additional immunological tool to mitigate the impact of *C. parvum* infections in neonatal calves.

## Figures and Tables

**Table 1:** Study vaccination schedule.

Category	Group	Vaccination—D0	Vaccination—D28
Pregnant cows/heifers	50% TEST	Bovilis Cryptium^®^ (2 mL SC) + Bovilis^®^ Rotavec^®^ Corona (2 mL IM)	Bovilis Cryptium^®^ (2 mL SC)
	50% CONTROL	Saline solution (2 mL SC) + Bovilis^®^ Rotavec^®^ Corona (2 mL IM)	Saline solution (2 mL SC)

**Table 2:** Number of cows by treatment group and Dutch dairy farm on day 0 (vaccination).

Farm	Control	Test
1	40	41
2	27	28
3	12	10
4	13	12
5	25	26
6	10	10
7	3	4
8	17	17
Total	147	148

**Table 3:** Number of cows by treatment group and French beef farm on day 0 (vaccination).

Farm	Control	Test
1	40	40
2	33	32
3	17	17
4	12	13
7	12	11
8	12	11
9	10	10
10	16	14
Total	152	148

**Table 4:** Faecal consistency score (FC).

Score	Description
0. Normal	Faeces retain form. May be pasty but do not flow across a tilted surface. Hindquarters are clean.
1. Mild diarrhoea	Semi-formed, thinner than usual, sufficient water to slowly flow across a tilted surface.
2. Moderate diarrhoea	Thickness of or tending towards syrup. Flows easily across or down across a tilted surface, while leaving some material, partly remains on top of bedding.
3. Severe diarrhoea	Watery faeces. Sifts through bedding.

**Table 5:** Percentage (%) of dairy calves scored with a faecal consistency score ≥ 2 from day 0 to 21 by treatment group.

	Treatment Group							
	CONTROL				TEST			
	Faecal Consistency Score > 0		Faecal Consistency Score ≥ 2 ^a^		Faecal Consistency Score > 0		Faecal Consistency Score ≥ 2 ^b^	
	No	Yes	No	Yes	No	Yes	No	Yes
	%	%	%	%	%	%	%	%
day	100.0	0.0	100.0	0.0	100.0	0.0	100.0	0.0
0								
1	98.6	1.4	99.3	0.7	97.1	2.9	99.3	0.7
2	95.8	4.2	99.3	0.7	94.2	5.8	97.8	2.2
3	94.4	5.6	96.5	3.5	97.1	2.9	99.3	0.7
4	95.1	4.9	99.3	0.7	98.6	1.4	100.0	0.0
5	95.7	4.3	98.6	1.4	97.0	3.0	98.5	1.5
6	89.4	10.6	93.7	6.3	91.1	8.9	94.1	5.9
7	73.2	26.8	83.1	16.9	74.5	25.5	83.9	16.1
8	56.3	43.7	69.0	31.0	70.1	29.9	79.6	20.4
9	49.3	50.7	67.6	32.4	57.7	42.3	73.0	27.0
10	47.9	52.1	66.2	33.8	49.3	50.7	71.3	28.7
11	54.2	45.8	71.1	28.9	53.7	46.3	76.5	23.5
12	62.0	38.0	83.1	16.9	58.1	41.9	81.6	18.4
13	74.5	25.5	92.9	7.1	71.3	28.7	90.4	9.6
14	79.4	20.6	92.9	7.1	75.7	24.3	92.6	7.4
15	85.1	14.9	93.6	6.4	83.1	16.9	91.2	8.8
16	87.1	12.9	95.0	5.0	82.4	17.6	95.6	4.4
17	87.1	12.9	92.9	7.1	85.3	14.7	97.8	2.2
18	88.6	11.4	92.9	7.1	87.5	12.5	97.8	2.2
19	88.6	11.4	95.7	4.3	91.2	8.8	98.5	1.5
20	91.4	8.6	97.9	2.1	93.4	6.6	99.3	0.7
21	94.3	5.7	97.1	2.9	97.0	3.0	98.5	1.5

^a,b^ odds ratio 1.25 [95% C.I.: 0.98, 1.61], *p*-value = 0.0770.

**Table 6:** Percentage (%) of suckling calves scored with a faecal consistency score ≥ 2 from day 0 to 21 by treatment group.

	Study Group							
	CONTROL				TEST			
	Faecal Consistency Score > 0		Faecal Consistency Score ≥ 2 ^a^		Faecal Consistency Score > 0		Faecal Consistency Score ≥ 2 ^b^	
	No	Yes	No	Yes	No	Yes	No	Yes
	%	%	%	%	%	%	%	%
Day of age	100.0		100.0		99.3	0.7	100.0	
0								
1	99.3	0.7	100.0		97.8	2.2	100.0	
2	98.6	1.4	98.6	1.4	98.6	1.4	99.3	0.7
3	98.6	1.4	99.3	0.7	98.6	1.4	100.0	
4	95.1	4.9	97.9	2.1	97.1	2.9	100.0	
5	95.1	4.9	97.9	2.1	97.1	2.9	99.3	0.7
6	94.4	5.6	97.2	2.8	97.8	2.2	99.3	0.7
7	91.7	8.3	95.8	4.2	97.8	2.2	98.6	1.4
8	90.2	9.8	95.1	4.9	95.0	5.0	97.1	2.9
9	89.5	10.5	92.3	7.7	92.1	7.9	97.8	2.2
10	87.4	12.6	94.4	5.6	89.9	10.1	95.0	5.0
11	88.1	11.9	96.5	3.5	85.6	14.4	95.0	5.0
12	83.9	16.1	92.3	7.7	84.9	15.1	93.5	6.5
13	84.6	15.4	99.3	0.7	87.1	12.9	94.2	5.8
14	88.1	11.9	97.2	2.8	89.9	10.1	98.6	1.4
15	90.9	9.1	97.2	2.8	90.6	9.4	97.1	2.9
16	89.5	10.5	97.2	2.8	89.9	10.1	97.1	2.9
17	90.2	9.8	97.2	2.8	89.2	10.8	97.8	2.2
18	91.6	8.4	97.9	2.1	89.2	10.8	95.7	4.3
19	90.2	9.8	98.6	1.4	87.1	12.9	96.4	3.6
20	93.0	7.0	98.6	1.4	89.9	10.1	95.7	4.3
21	93.0	7.0	96.5	3.5	94.2	5.8	96.4	3.6

^a,b^ Odds Ratio 1.12 [95% C.I.: 0.71, 1.77], *p*-value = 0.6147.

**Table 7:** Summary statistics of duration of diarrhoea, specified as the number of days with a faecal consistency (FC) score of ≥2 by treatment group, for all dairy calves.

	N	Mean Number of Days	Std	Min	Q1	Median	Q3	Max
CONTROL	143	2.2	2.1	0.0	0.0	2.0	3.0	8.0
TEST	140	1.8	1.8	0.0	0.0	1.0	3.0	9.0

Mixed model ANOVA: Estimate [95% CI] control vs test = 0.4527 [0.0441, 0.8613], *p*-value = 0.0300.

**Table 8:** Summary statistics of duration of diarrhoea, specified as the number of days with a faecal consistency (FC) score of ≥ 2 by treatment group, for all suckling calves.

	N	Mean Number of Days	Std	Min	Q1	Median	Q3	Max
CONTROL	144	0.6	1.1	0.0	0.0	0.0	1.0	6.0
TEST	139	0.6	1.1	0.0	0.0	0.0	1.0	6.0

**Table 9:** Number and percentages of calves with their maximum faecal consistency score.

Max FCS		Control		Test	
		N	%	N	%
Score 0	Dairy	21	14.7	28	20.0
	Suckling	75	52.1	78	56.1
Score 1	Dairy	20	14.0	20	14.3
	Suckling	25	17.4	22	15.8
Score 2	Dairy	59	41.3	61	43.6
	Suckling	24	16.7	24	17.3
Score 3	Dairy	43	30.1	31	22.1
	Suckling	20	13.9	15	10.8
All	Dairy	143		140	
	Suckling	144		139	

**Table 10:** Number and percentage (%) of test results in the *C. parvum* test by result and study group.

		Control		Test	
		N	%	N	%
Negative	Dairy	17	16	16	18
	Suckling	23	50	28	62
Weak positive	Dairy	12	12	16	18
	Suckling	4	8	2	4
Positive	Dairy	75	72	57	64
	Suckling	19	41	15	33
Total tests (*n* = total number of calves in the group)	Dairy	104 (*n* = 143)		89 (*n* = 140)	
	Suckling	46 (*n* = 144)		45 (*n* = 139)	

**Table 11:** Numbers of treatments in calves by indication and study group.

Treatment Group					
		Control		Test	
		N	%	N	%
Diarrhoea	Dairy	85	58.6	73	49.3
	Suckling	26	18.1	21	15.1
Respiratory disease	Dairy	1	0.7	1	0.7
	Suckling	5	3.5	1	0.7
General internal disease	Dairy				
	Suckling	3	2.1	7	5.0
Locomotion	Dairy				
	Suckling	1	0.7		
Renal/omphalitis	Dairy				
	Suckling	14	9.7	17	12.2
Other	Dairy	9	6.2	11	7.4
	Suckling				

**Table 12:** Dairy and suckling calves: Descriptive statistics for the antibody titres measured in colostrum and serum by treatment group.

log2Ab Titre		Control			Test		
		N	Mean	SD	N	Mean	SD
Colostrum	Dairy	138	14.9	1.5	140	19.6	0.8
	suckling	140	15.7	1.8	137	19.5	1.2
Serum	Dairy	142	11.3	1.6	136	18.1	1.7
	Suckling	143	12.1	2.0	139	17.2	2.4

## Data Availability

Data are contained within the article.
